# Self-Powered Fine Dust Filtration Using Triboelectrification-Induced Electric Field

**DOI:** 10.1186/s11671-022-03749-6

**Published:** 2022-12-23

**Authors:** Young-Jun Kim, Hyoung Taek Kim, Jeong Hwan Lee, In-Yong Suh, Sang-Woo Kim

**Affiliations:** 1 School of Advanced Materials Science and Engineering, Sungkyunkwan University (SKKU), Suwon, 16419 Republic of Korea; 2 SKKU Advanced Institute of Nanotechnology (SAINT), SKKU Institute of Energy Science and Technology (SIEST), Sungkyunkwan University (SKKU), Suwon, 16419 Republic of Korea

**Keywords:** Triboelectric nanogenerator, Particulate matter, Air quality, Fine dust filtration, Self-powered operation

## Abstract

Particulate matter (PM) in the environment can adversely affect the health of living things. However, high removal efficiency and low-pressure loss are crucial design challenges for any air filtration system. To circumvent the challenge, here, we demonstrate a novel triboelectric (TE) air filtration system that is based on a rotation-type triboelectric nanogenerator (TENG) and a filter comprising two sets of plates: primary and secondary, that are placed in the airflow channel. When the TENG charges the two plate sets with opposite charges, the flowing air particles are charged at the primary plates and are collected, due to an electric field, at the secondary plates. The TE filter has demonstrated a PM_2.5_ removal efficiency of ~ 99.97% for the fine dust particles, and it remains stable even after several washing cycles. The pressure loss is almost two orders less than the high-efficiency particulate air filter. Since the airflow itself can drive the TENG, the TE filter can potentially be integrated with any air conditioning system for fine dust filtration in offices, automobiles, etc.

**Supplementary Information:**

The online version contains supplementary material available at 10.1186/s11671-022-03749-6.

## Introduction

The rising air pollution is posing a great danger to the health of all living things and the environment. In fact, various industrial processes such as fossil fuels combustion for energy production, burn forming, construction, etc. produce, as a byproduct, particulate matters (PMs) that inevitably contaminate the atmosphere [1–4]. PMs can comprise various chemicals such as organic carbon, chloride, nitrate, sulfate, iron, elemental carbon, and calcium. Regarding size, PMs can be very small and their dimension can range from several nanometers to micrometers [5, 6].

PM_10_ has dimensions in the range of 2.5 µm to 10 µm, while PM_2.5_ are fine particles with dimensions less than 2.5 µm. PMs are inhalable and can potentially penetrate human bronchi and lungs [7]. For instance, exposure to PM_2.5_ can result in morbidity and mortality due to respiratory and cardiovascular diseases [2, 6]. Besides, it is also suggested that PM_2.5_ exposure could also cause other diseases such as asthma, chronic obstructive pulmonary disease, pulmonary fibrosis, cancer, type 2 diabetes, and neurodegenerative diseases [1, 3]. Ultrafine particles (UFP), on the other hand, with dimensions less than 0.1 µm, i.e., PM_0.1_, pose an even greater danger to public health as they have a higher concentration, higher surface area-to-mass ratio, and higher chemical reactivity [1, 6]. As a matter of fact, UFPs can cause serious issues to human health such as ventriculomegaly, neurochemical disruption, glial activation preferentially, and stroke damage exacerbation. In general, PMs can adversely influence air quality. Recently, fine dust with dimensions less than 10 µm is regarded as a grave danger, and the development of air filtration systems is highly desired.

Two of the main challenges in the design of air filtration systems are high removal efficiency and low airflow resistance or, in other words, low-pressure drop. Electrostatic precipitation is one method for PMs filtration, but, due to high electric fields involved, it is subject to air ionization producing ozone which can also cause damage to human health. On the other hand, fibrous filters, such as high-efficiency particulate air (HEPA) filters and polymer nanofiber films, can very efficiently remove the PMs that are larger than the holes within the filter. However, their efficiency can be significantly affected for PMs smaller than the holes, such as UFPs [8]. Besides, fibrous filters, due to their structure, inherently result in pressure drop [1, 6].

Triboelectric nanogenerators (TENGs) are proposed for mechanical energy harvesting with the crucial advantages of structural simplicity, low cost, and robustness [9, 10]. Their operation is based on contact electrification and electrostatic induction [11]. TENGs have a typically very high open-circuit voltage (*V*_*oc*_) [12–14]. TENGs-based air filters were recently introduced for the removal of various PMs. In fact, the TENG-based filter had demonstrated excellent particle removal efficiency (~ 90%), but the TE filter, due to the design involving the number of PTFE and nylon fabrics layers or multilayered polyimide (PI) nanofiber, could potentially suffer from pressure loss. Recently, the removal of airborne microbes through a filter based on TENG was reported [15]. In the work, the airborne microbes were inactivated in 2 steps: i) contact electrification and ii) inactivating by electroporation after collecting them by electric field.

Here, we introduce a TENG-based air filtration system which mainly comprises a rotation-type TENG, and a filter technology based on our previous work [15]. The TENG has a stator with nylon and polytetrafluoroethylene (PTFE) as friction layers, placed side by side, and a rotator with Kapton as the friction layer with Cu/Au-based back electrodes. The filter is based on two sets of plates, placed side by side. The primary plates are a set of Al plates, while the secondary plates consist of a set of polyvinylidene difluoride (PVDF)-coated Al plates with additional grounded Al plates cascaded in between the secondary plates. Due to the high dielectric properties of PVDF, no air breakdown occurred, and consequently, no ozone was generated [16, 17]. Unlike fiber and fabric-based filters [17], this filter has much lower air resistance and can potentially undergo a much lower pressure loss. When the TENGs output is applied across the filter, the particles in the air are charged at the primary plates and, due to the electric field, are absorbed at the secondary plates. Remarkably, the TENG-based air filtration system does not require any external power as the TENGs are driven by the airflow itself. It can therefore be integrated with various air conditioning systems for air purification in various environments such as offices, automobiles, and houses.

## Experimental

### Fabrication of TENG

Generating parts are divided into two parts. First, in the case of the stator bottom plate, the PI film substrate comprises of negative, positive electrodes connected to each part, negative charge and positive charge collectors, and there is an independent neutral bar. Each electrode is protected by PI film. 80 nm of Au/18 μm of Cu is deposited on 100 μm films. Fabrication is made according to the general flexible printed circuit board (FPCB).

### Charging, Collector Parts

In the case of the charging part, an Al plate is formed. The angle of each plate is 45 degrees and is installed at an interval of 2 cm. In the case of collection parts, parallel plates were arranged at intervals of 5 mm and were all formed using a particle collection plate and a ground plate repeatedly. In the case of the particle collection plate, the PI film adheres on both sides to prevent discharge. Each of these parts is connected to the negative and positive grounds to perform their respective roles.

### Filtering Tests and Characterization

First, the test was carried out in a box with a closed space of 800 mm × 1500 mm × 300 mm. This space modeled the space where actual air was purified. In addition, a small air conditioner-type structure was formed in a box of 210 mm × 510 mm × 210 mm to form an air blower, a filter, and a sensor. To make the particulate matter, A2 test dust (ISO 12103–1, PTI) is crushed into a rotating plate and flowed to the air blower through blowing. For efficiency measurement, the value is attained through the particle counter at the inflow side, and the efficiency is calculated by measuring particle dust flowing through the TE filter with a particle counter. A DPO 3052 digital phosphor oscilloscope (Tektronix Inc., Beaverton, OR) was used to detect the open-circuit voltage signals, and a low-noise current amplifier (Model SR570, Stanford research systems) was used to detect the short-circuit current signals that were generated by the generator part. And the fine dust of the collection part was confirmed through optical microscopy (SAMWON). The number of particles passing through the filter was measured through the particle counter (Handheld, HH3016). The ozone concentration in the air was measured by an ozone meter (Model T400, Teledyne API, Inc.

## Results and Discussion

**Fig. 1:**
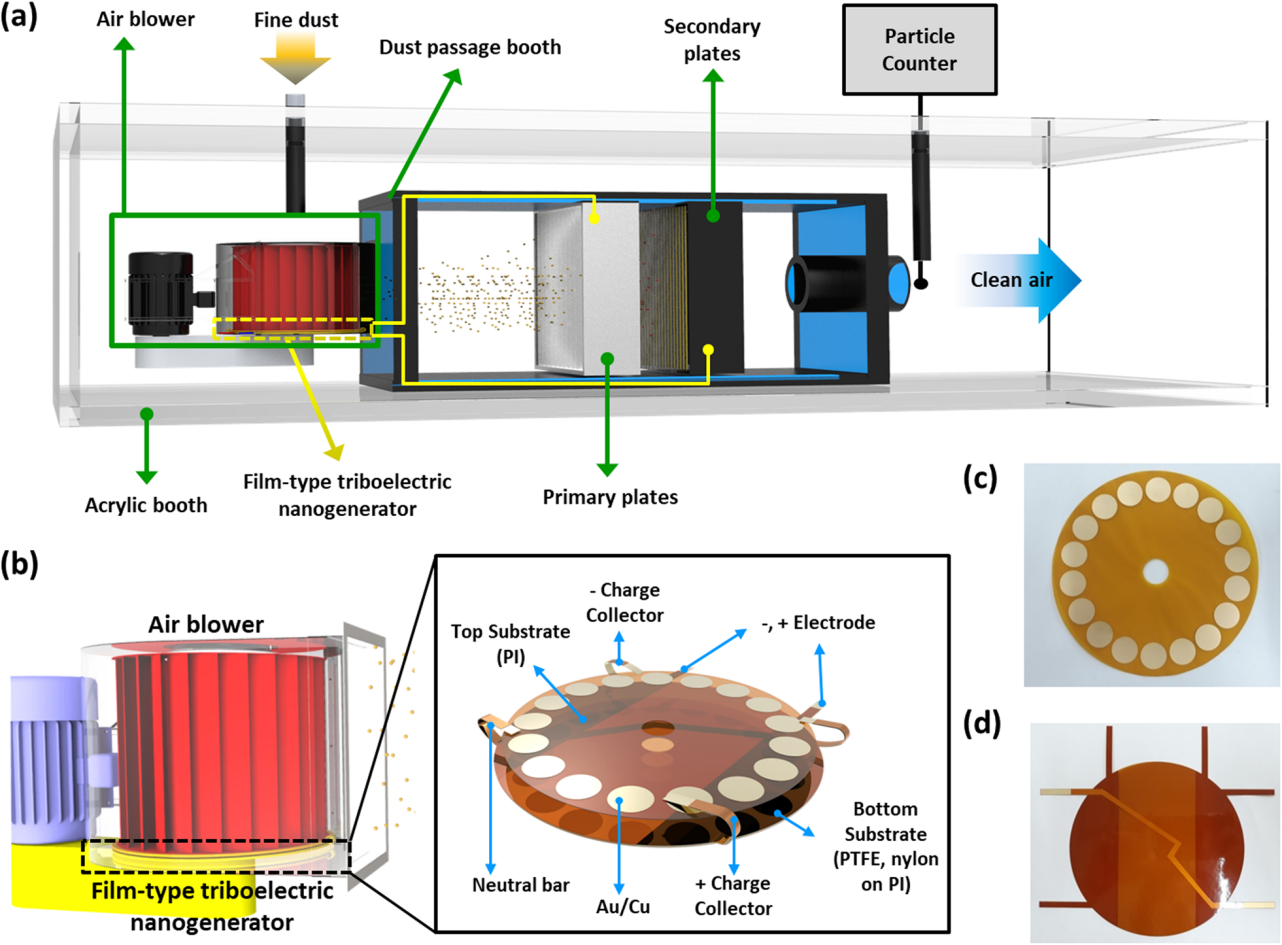
**a** Schematic description of the TENG-based fine dust filtration system. **b** Schematic design of the rotation-type TENG. **c** and **d** As fabricated stator and the rotator of the TENG

Figure 1A schematically represents the TENG-based fine dust filtration system. Initially, an electric motor-driven air blower creates airflow. The rotation-type TENG was mounted on top (see Fig. 1b) of the air blower. The filter is placed in the air channel and comprises two sets of plates: The primary plates comprise Al plates; the secondary plates comprise cascaded Al and PVDF-coated Al plates (for details on the filter, see Fig. 3 and discussion). Fine dust is incorporated into the air from a channel above the blower. The fine dust-contaminated air is then injected into a chamber that is equipped with a filter. There is also a particle counter at the exit of the airflow to count the fine dust particles as a measure of performance of the filtration system. Figure 1 b describes the TENG schematic design. The TENG, which is mounted on the air blower, is based on a rotation-type TENG, and it consists mainly of two parts: stator and rotator. The stator comprises PTFE and nylon friction layers, placed side by side, in two same-size sectors on a Kapton substrate which is a part of the FPCB. The rotator, on the other hand, is based on a flexible PCB Kapton substrate with circular disk-shaped electrodes on top. The electrodes are realized by patterning the Cu layer (of the PCB) in the shape of circular disks. To prevent oxidation in the working environment and to reduce friction, the Au layer is deposited on the Cu layer. There are N electrodes, i.e., N/2 per friction layer. The TENG requires two additional, crucial connections. The first one is the “neutral bar,” which is realized on the backside of the stator by patterning the Cu film (of the flexible PCB). The second is the “charge collectors,” which also comprise Cu patterns that are realized on the back side of the stator, but they are insulated from the neutral bar; the insulation is achieved by Cu patterning on an additional flexible PCB and contacting it, beneath the neutral bar, to the back of the stator. Both neutral bar and charge collectors, at the edges, are bent upward and make a connection with the top electrodes of the rotator during rotations. The charge collectors serve as the output terminals as they apply the TENG’s output to the filter. Figure 1c, d shows the fabricated rotator and stator of the TENG, respectively, and the fabrication process is described in detail in Experimental section.

**Fig. 2:**
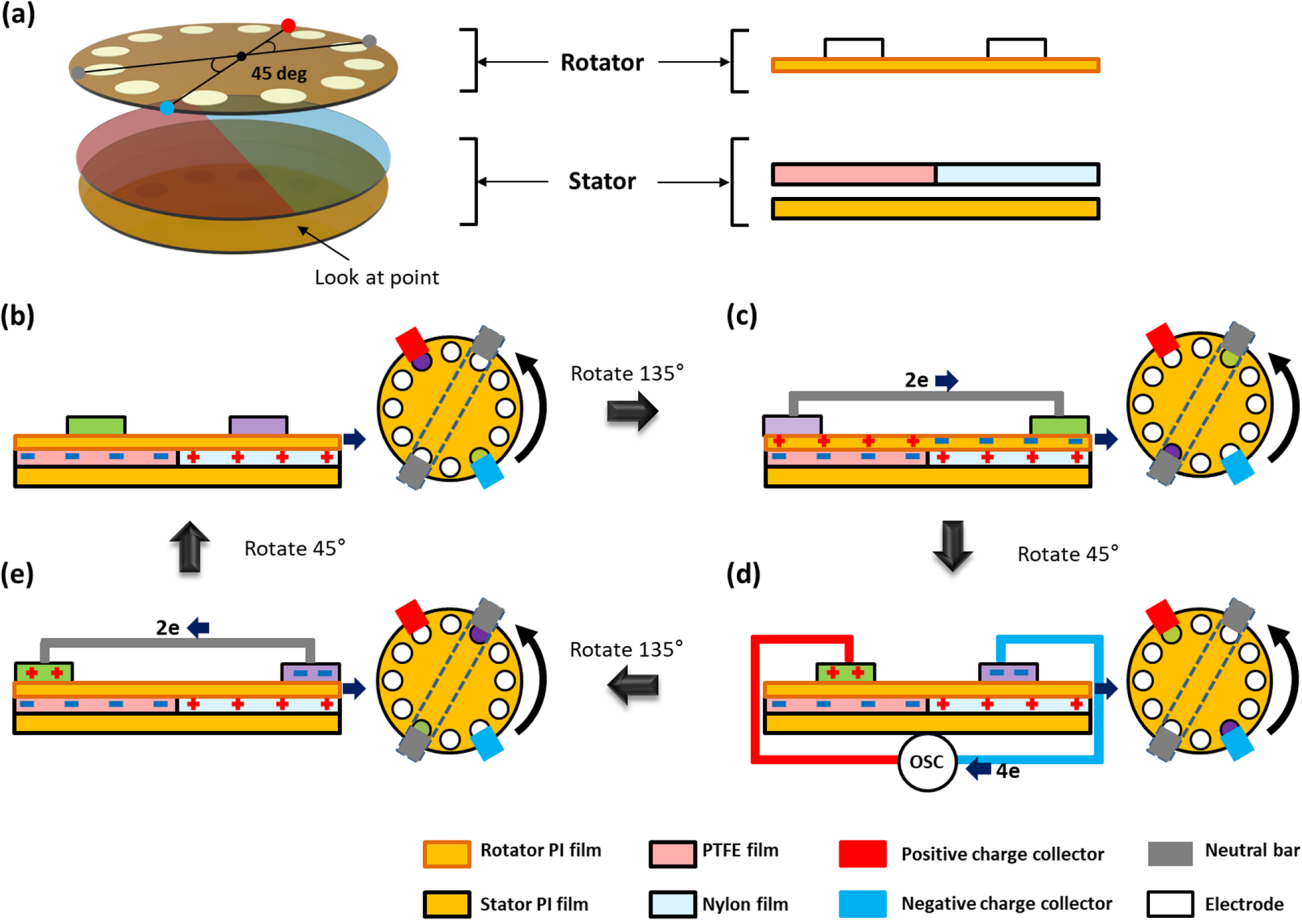
The working mechanism of the TENG. **a** Schematic description of the film-type TENG. **b**–**e** Schematic illustration showing the suggested working mechanism of the film-type TENG with electron flow diagrams

The working mechanism of the TENG is schematically described in Fig. 2. Figure 2a shows a schematic image of TENG and its cross-sectional view. Initially, due to the rotational friction (see Fig. 2a), PTFE’s surface has negative charges, nylon’s surface has positive charges (Fig. 2b), whereas the Kapton’s surface can potentially have both positive and negative charges as it contacts both PTFE (which is triboelectrically more negative [18, 19]) and nylon (which is triboelectrically more positive [18, 20]). As a consequence, the charges on the opposite sides of the frictional contact do not fully screen each other, which potentially results in electrostatic induction in the electrodes. Since we made rotation mode TENG, we used flat film as friction layers (Additional file 1: Fig. S1). Along the rotational motion, when the two circular disk electrodes (Fig. 2c) on the opposite side are short-circuited by coming into contact with the neutral bar, there is a charge transfer between them due to the electrostatic induction. After a rotating the rotator by 45°, the two electrodes move from one friction layer to another [21–24] and come into contact with the charge collector and consequently, there is a charge transfer (Fig. 2d). After a further rotating by 135°, the two electrodes depart the charge collector and again come into with neutral bar, and there is a charge transfer between them (see Fig. 2e). To complete the cycle, a further rotating by 45° brings the two electrodes into contact with the charge collector again, but, in this orientation, they again move from one friction layer to another and, as a result, there is a charge transfer between them in the opposite direction. In brief, the neutral bar facilitates charge transfer between the opposite electrodes under short-circuit conditions and the charge collector facilitates charge transfer between the electrodes, in the opposite direction, through the filter.

**Fig. 3:**
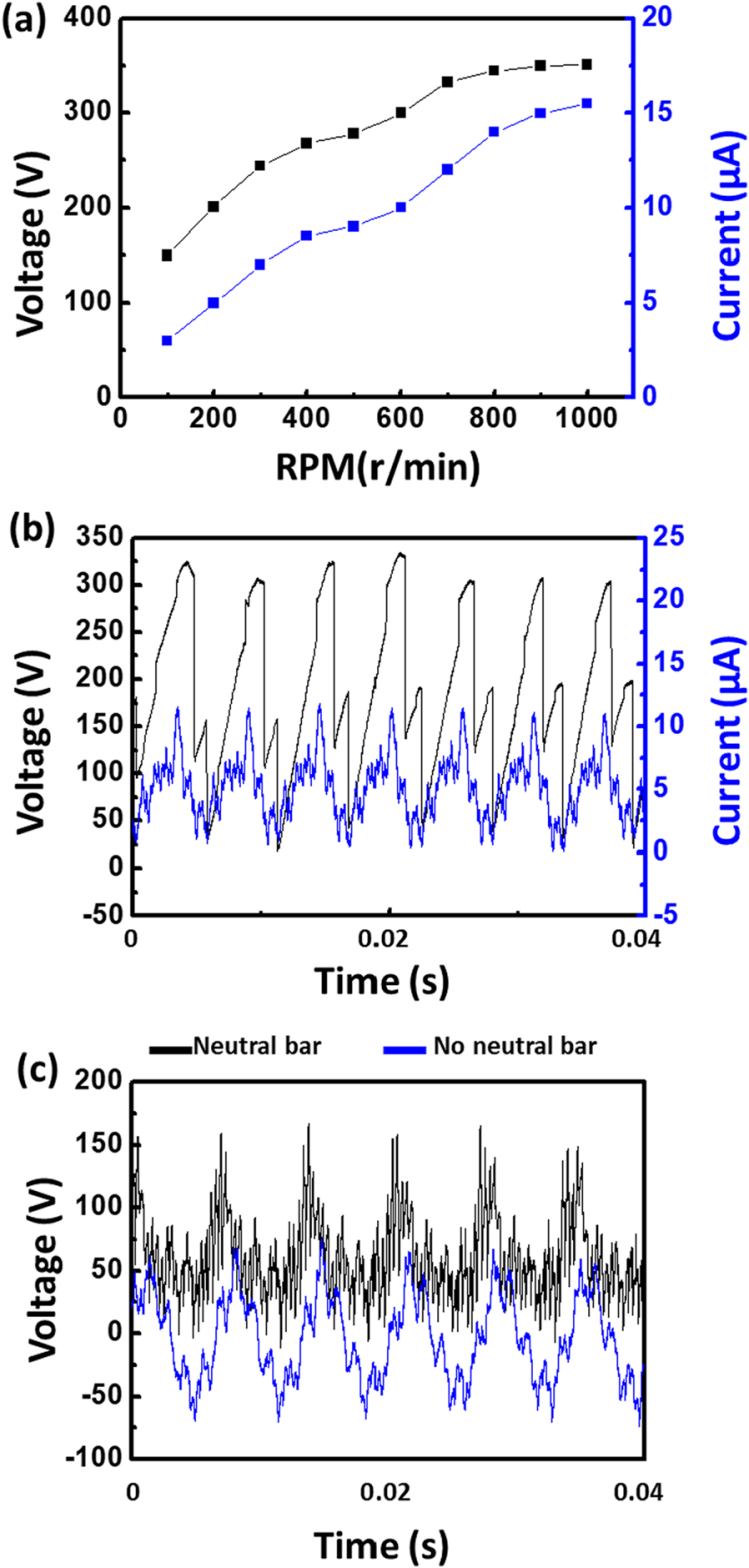
Performance characterization of the rotation-type TENG. **a** The output voltage and current of the TENG as a function of rotation. **b** Time evolution of the TENG’s output at 800 rpm. **c** TENG’s output voltage with and without the neutral bar

We carried out the performance characterization of the rotation-type TENG, while it was operated with air blower (see Fig. 3). Figure 3a shows the peak output voltage and current as a function of the rotational speed. It can be seen that the output voltage and current increase with the rotation speed. Figure 3b shows the time evolution of the TENG’s output voltage and current at 800 rotations per minute (rpm). The TENG has a high output voltage with a peak output voltage of 335 V. The thickness of the rotor PI film was changed to 10, 25, 50, and 100 μm to check the output power according to the film thickness (Additional file 1: Fig. S2). The output power of the TENG increases as the thickness decreases, but on the contrary, when the film thickness becomes too thin, the output power of the TENG decreases due to the leakage current. Since the neutral bar plays a key role in providing direct current (DC) output, we also characterized the TENG’s output with and without the neutral bar (see Fig. 3c). The output voltage of the TENG with neutral bar is DC, whereas the output voltage without neutral bar is AC.

**Fig. 4:**
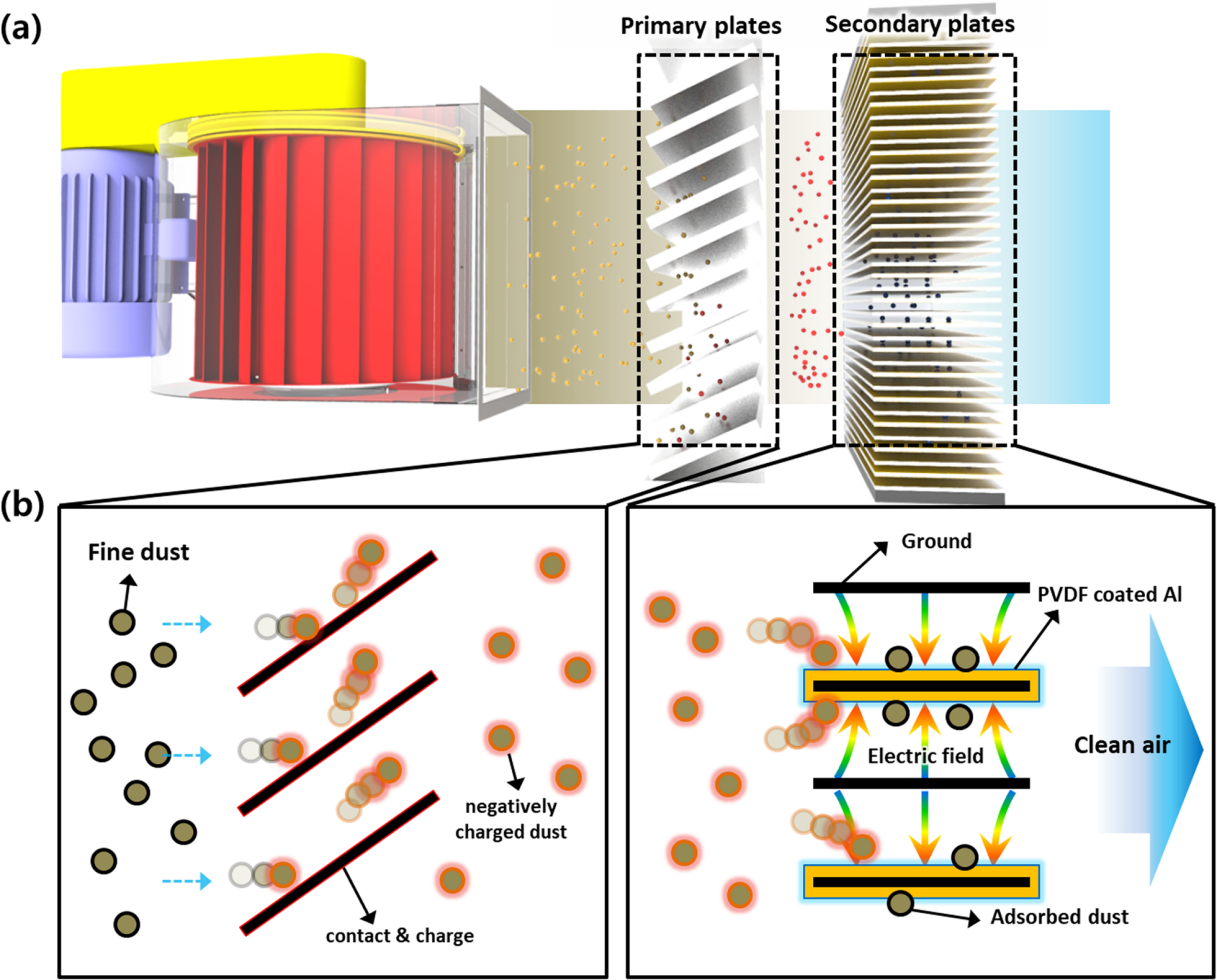
Schematically described structure **a** working mechanism of the fine dust filtration system. **b** Illustrations of the TE filter in the fine dust filtration system. It consists of a bare Al plate charge supplying negative Al electrodes and integrated positive (PVDF-coated Al)/ground (Al) electrodes. In charge supplying part, fine dust contact with the charge supplying Al electrode, and the fine dust negatively charged. In the dust collecting part, the negatively charged fine dust is attracted to the positive electrode by the Coulomb force by the electric field generated between the positive electrode and the ground electrode

Figure 4 schematically describes the structure and working mechanism of the fine filtration of the fine dust. The TENG’s output is applied across the two sets of plates such that the primary plates are negatively charged, and the secondary plates are positively charged. To prevent ozone generation, PVDF with high dielectric constant (> 12), i.e., high energy storage capacity based on ferroelectric properties, was used (Additional file 1: Fig. S3 and S4). As mentioned earlier, the secondary plates have grounded Al plates inserted between the PVDF-coated Al plates. As a consequence, an electric field is created from the positively charged PVDF-coated plates to the grounded Al plates. Therefore, under the influence of the airflow, when the dust particles pass through the primary plates, they inevitably contact the Al plates due to their orientation and are therefore negatively charged. When they pass through the secondary plates, being negatively charged, they move against the electric field and are deposited at the positively charged PVDF-coated Al plates. Due to the adsorption of various sizes fine dust particles (Additional file 1: Fig. S5), the air exiting the filter is clean [25, 26].

**Fig. 5:**
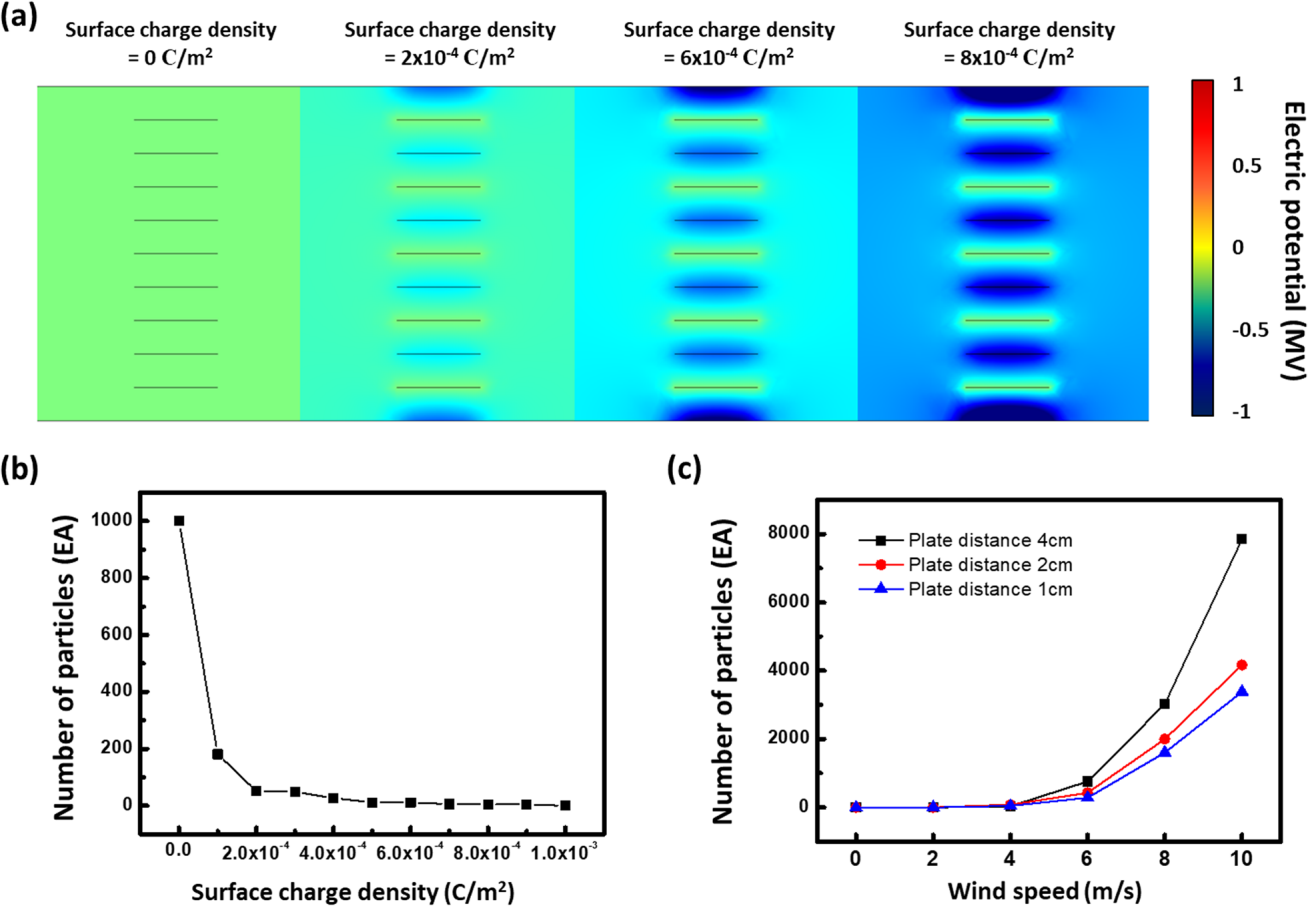
COMSOL simulation of the dust collection at the secondary plates of the filter for air filtration. **a** The electric potential in the secondary plates, which is the dust collecting part, for an increasing surface charge density at the PVDF-coated Al plates. **b** The number of particles getting through the secondary plates without filtration as a function of the surface charge density. **c** The number of particles getting through the secondary plates without filtration as a function of the particle velocity at various plate spacing

To investigate crucial parameters of the dust filtration process, we carried out COMSOL simulation of the secondary plates (simulation conditions are shown in Additional file 1: Fig. S6), which is the dust collection part, of the filter using the electrostatic module. Charged particles with a charge density of 0 to 8 × 10^–4^ μC/cm^2^ were moved through the secondary plates; the Al plates were at ground potential, whereas the PVDF-coated Al plates had a surface charge. Figure 5a shows that the electric potential field is strongly related to an increased surface charge density at the secondary plates. It is evident that the electric potential field increases as the surface charge density increases, thereby increasing the potential for dust adsorption. Figure 5b shows the number of particles going through, i.e., without being adsorbed at, the secondary plates as a function of the surface charge density. It can be seen that as the surface charge increases, the number of particles escaping the filter decreases. Figure 5c indicates that wind speed and plates spacing are also crucial as the number of particles escaping the secondary plate increases as the plate spacing and wind speed increase.

**Fig. 6:**
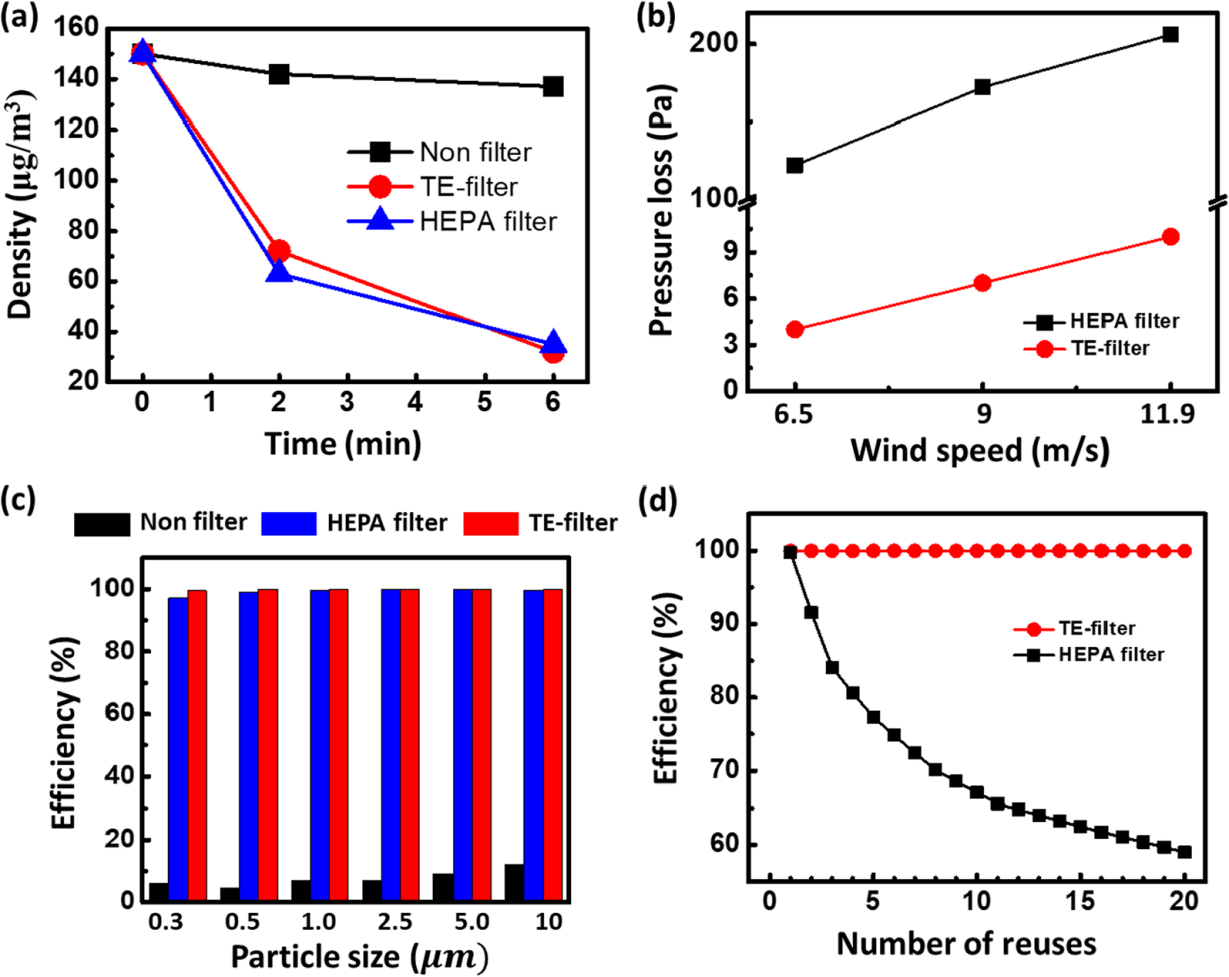
Performance characterization of TE filter. **a** Performance comparison, in terms of the density of the particles in the filtered air, of the proposed filter with the commercial HEPA filter and with the case of no filter. **b** Comparison of the pressure loss due to the presence of the TE filter with that of the HEPA filter. **c** Dust collection efficiency as a function of the particle size for the proposed TE filter, HEPA filter, and the case of no filter. **d** Change in dust collection efficiency after washing for both HEPA filter and TE filter

To investigate the performance of the dust filtration system, we carried out detailed experimental characterizations and the results are shown in Fig. 6. Dusts with PM_0.3_ and PM_10_ were used to evaluate the filtering performance. The TE filter, i.e., TENG-based filtration system, efficiently reduces the density of the particles with time (Additional file 1: Fig. S7) in comparison with the case of no filter, and besides the filtration performance is similar to the commercial HEPA filter (see Fig. 6a). It takes 8 min for the TE filter to completely remove fine dust. Therefore, we confirmed that TENG is working normally during that time. The results show that the output performance of TENG is maintained for 8 min (Additional file 1: Fig. S8). Figure 6b shows that the TE filter is superior to HEPA filter as it undergoes relatively lower pressure loss two orders lower than the HEPA filter. Particle size is a crucial factor that influences the efficiency of a filter. Figure 6c shows the dust collection efficiency as a function of the particle size for the TE filter, HEPA filter, and no filter case. TE filter has shown a higher efficiency than the HEPA filter, particularly for smaller particle sizes (~ 3 µm). Since the particles are adsorbed by the filter, after a certain time, it requires washing for further use. The results show that particles having a size of 2.5 μm and a size of 10 μm were captured with 99.7% and 100%, respectively. In addition, to verify whether ozone is generated during the operation of the TE filter, the ozone concentration in the air around the filter was measured for 1 h. As a result of the measurement, the maximum ozone concentration in the air was less than 0.001 μmol/mol, which was not different from before the TE filter was operated. Accordingly, Fig. 6d shows a dust collection efficiency comparison of the TE filter and the HEPA after a number of washing cycles. It is evident that the efficiency of the TE filter remains stable after washing, whereas the efficiency of the HEPA filter significantly deteriorates due to washing. Performance characterization demonstrates that the TE filter presents a more stable, effective, and high-efficiency method with much lower pressure for fine dust filtration.

## Conclusions

We have demonstrated a TE filter for the filtration of fine dust from the air. The TE filter comprises a rotation-type TENG and a filter. The filter comprises two sets of plates: primary and secondary, and is placed in the airflow channel. When the TENG charges the two plates set with opposite charges, the flowing air particles are charged at the primary plates and are collected, due to an electric field, at the secondary plates. The TENG produces a high output voltage of ~ 335 V at an rpm of 800. The high output voltage from the TENG is crucial as the COMSOL simulation has shown that the number of particles that pass through without filtration decreases with increasing surface charge density at the secondary plates. Despite the high voltage, ozone was not generated due to the high dielectric constant of PVDF film. With a removal efficiency of ~ 99.7% for PM_2.5_, TE filter has comparable removal efficiency to that of HEPA filter. Remarkably, the pressure loss is almost two orders less than that of the HEPA filters. Besides, after several washing cycles, its removal efficiency remains stable, whereas the removal efficiency for the HEPA filter drops sharply. TENG can be driven by the airflow itself, and therefore, the TE filter is suitable for integration with air conditioning systems for air purification in offices, homes, automobiles, etc.

## Supplementary Information

**Additional file 1**. **Fig S1** SEM images showing surface morphologies of each friction layer of TENG (scale bar = 2 μm). **Fig S2** Output voltage of TENG depending on the thickness of the rotator PI film at a load resistance of 40 MΩ. **Fig S3** SEM image for PVDF with a thickness of 7 μm coated on an Al plate. The sample were prepared by bar-coating on Al/Si substrate and heat treated at 180 °C for 2 h to get β-phase PVDF films. Scale bar indicates 10 μm. **Fig S4** Polarization-Field (P-E) hysteresis curve for the sample shown in Fig. S2. **Fig S5** OM images of the PVDF-coated Al plate after collecting the fine dust. This result shows that PMs of various sizes were adsorbed to the PVDF coated Al plate by electrostatic attraction. **Fig S6** FEM simulation conditions for collection efficiency of fine dust particles according to surface charge density and flow velocity in the air duct. Bare Al and PVDF-coated Al were placed at intervals of 2 cm in a 20 cm-sized air duct, and bare Al was connected to the ground. 0, 2 × 10, 4 × 10, 6 × 10, and 8 × 10 C/m^2^ of surface charge density were applied to PVDF-coated Al, and the velocity of fine dust was set to 0, 2, 4, 6, 8, and 10 m/s. **Fig S7** Experimental data of PM_2.5_ level that were measured by a dust detector. The PM sensor shows that the PM value decreases significantly from 166 to 11 μg/m^3^ while the TENG drives for 8 min. **Fig S8** Output voltage of TENG for 8 minutes at a load resistance of 40 MΩ.

## Data Availability

All data supporting the conclusions of this article are included within the article.

## Abbreviations

PM: Particulate matter
TE: Triboelectric
TENG: Triboelectric nanogenerator
UFP: Ultrafine particles
HEPA: High-efficiency particulate air
PTFE: Polytetrafluoroethylene
PI: Polyimide
PVDF: Polyvinylidene difluoride
FPCB: Flexible printed circuit board
PCB: Printed circuit board
