# Supplementary material for: Self-Powered Fine Dust Filtration Using Triboelectrification-Induced Electric Field
Source: Nanoscale Res Lett. 2022 Dec 23;17:128. doi: 10.1186/s11671-022-03749-6 (PMC9789239; doi:10.1186/s11671-022-03749-6)
Supplement: Supplementary file 1 — Additional file 1. Fig S1 SEM images showing surface morphologies of each friction layer of TENG (scale bar = 2 μm). Fig S2 Output voltage of TENG depending on the thickness of the rotator PI film at a load resistance of 40 MΩ. Fig S3 SEM image for PVDF with a thickness of 7 μm coated on an Al plate. The sample were prepared by bar-coating on Al/Si substrate and heat treated at 180 °C for 2 h to get β-phase PVDF films. Scale bar indicates 10 μm. Fig S4 Polarization-Field (P-E) hysteresis curve for the sample shown in Fig. S2. Fig S5 OM images of the PVDF-coated Al plate after collecting the fine dust. This result shows that PMs of various sizes were adsorbed to the PVDF coated Al plate by electrostatic attraction. Fig S6 FEM simulation conditions for collection efficiency of fine dust particles according to surface charge density and flow velocity in the air duct. Bare Al and PVDF-coated Al were placed at intervals of 2 cm in a 20 cm-sized air duct, and bare Al was connected to the ground. 0, 2 × 10, 4 × 10, 6 × 10, and 8 × 10 C/m2 of surface charge density were applied to PVDF-coated Al, and the velocity of fine dust was set to 0, 2, 4, 6, 8, and 10 m/s. Fig S7 Experimental data of PM2.5 level that were measured by a dust detector. The PM sensor shows that the PM value decreases significantly from 166 to 11 μg/m3 while the TENG drives for 8 min. Fig S8 Output voltage of TENG for 8 minutes at a load resistance of 40 MΩ. [file 11671_2022_3749_MOESM1_ESM.docx]

**Supplementary Information (SI)**

**Self-Powered Fine Dust Filtration Using Triboelectrification-Induced Electric Field**

*Young-Jun Kim^a^*†, *Hyoung Taek Kim^a^*†, *Jeong Hwan Lee^a^*†*, In-Yong Suh^a^,*

*and Sang-Woo Kim^a,b,^**

^a^ School of Advanced Materials Science and Engineering, Sungkyunkwan University (SKKU), Suwon, 16419, Republic of Korea.

^b^ SKKU Institute of Energy Science and Technology (SIEST), SKKU Advanced Institute of Nanotechnology (SAINT), Sungkyunkwan University (SKKU), Suwon, 16419, Republic of Korea.

†These authors contributed equally to the work.

*Corresponding authors: E-mail: kimsw1@skku.edu


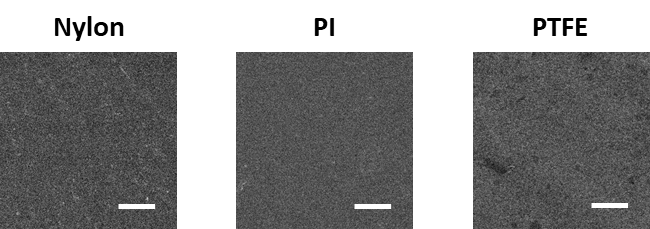


**Figure SI-1 │**SEM images showing surface morphologies of each friction layer of TENG (scale bar = 2 μm).

*
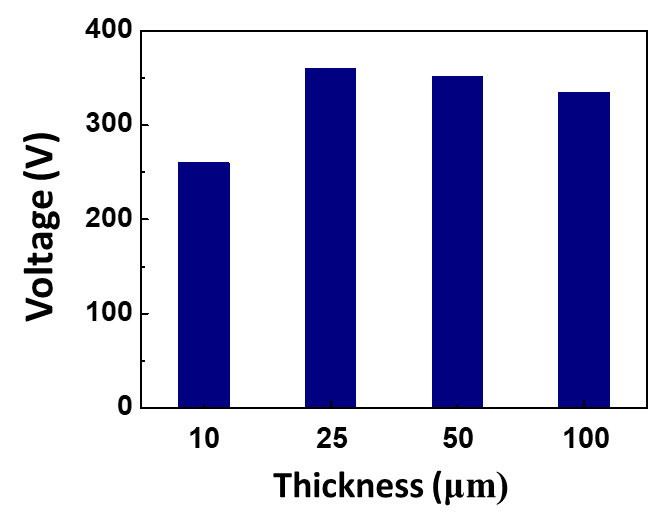
*

**Figure SI-2│** Output voltage of TENG depending on the thickness of the rotator PI film at a load resistance of 40 MΩ.

**
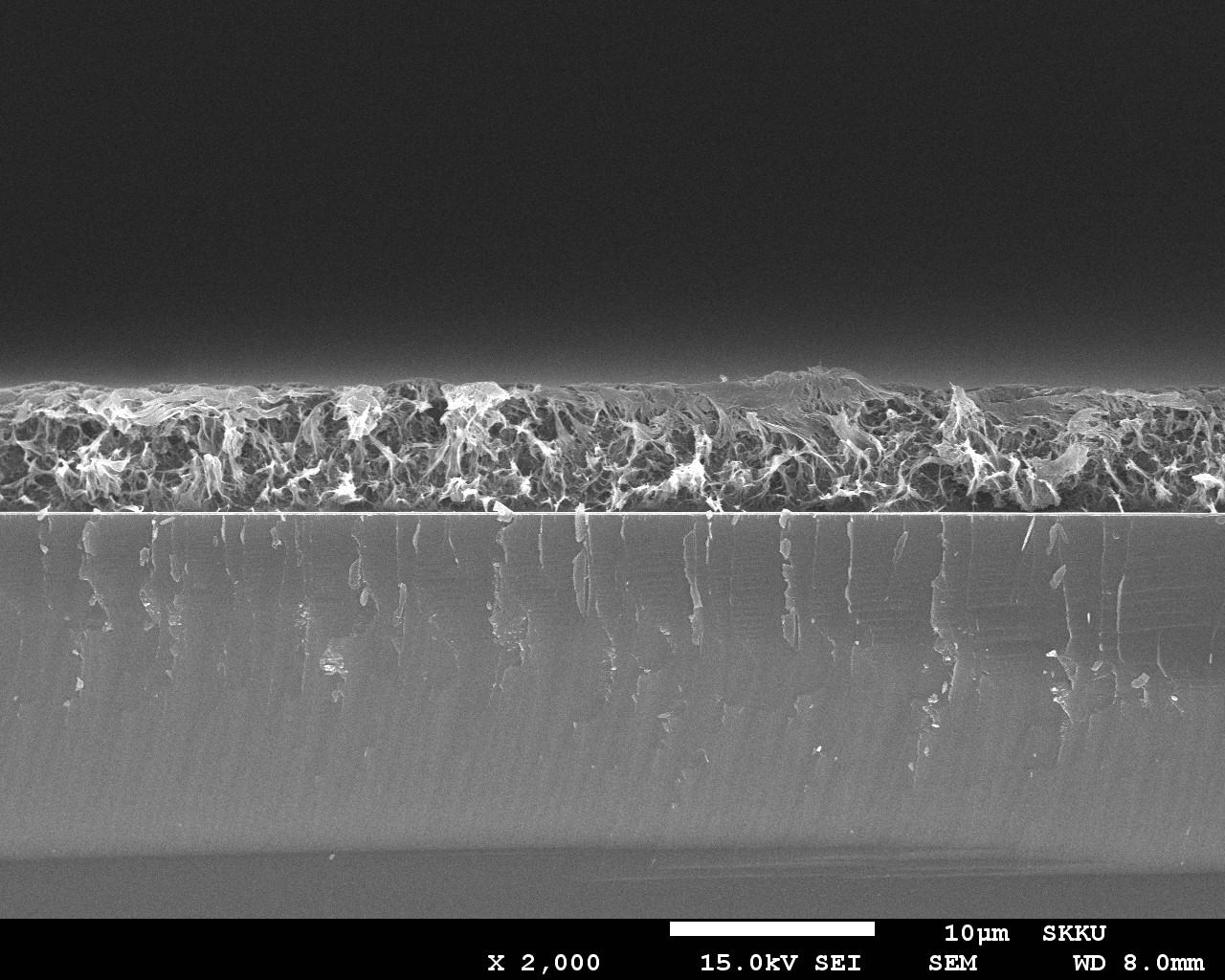
**

**Figure SI-3│** SEM image for PVDF with a thickness of 7 μm coated on an Al plate. The sample were prepared by bar-coating on Al/Si substrate and heat treated at 180 ^o^C for 2 hours to get β-phase PVDF films. Scale bar indicates 10 μm.


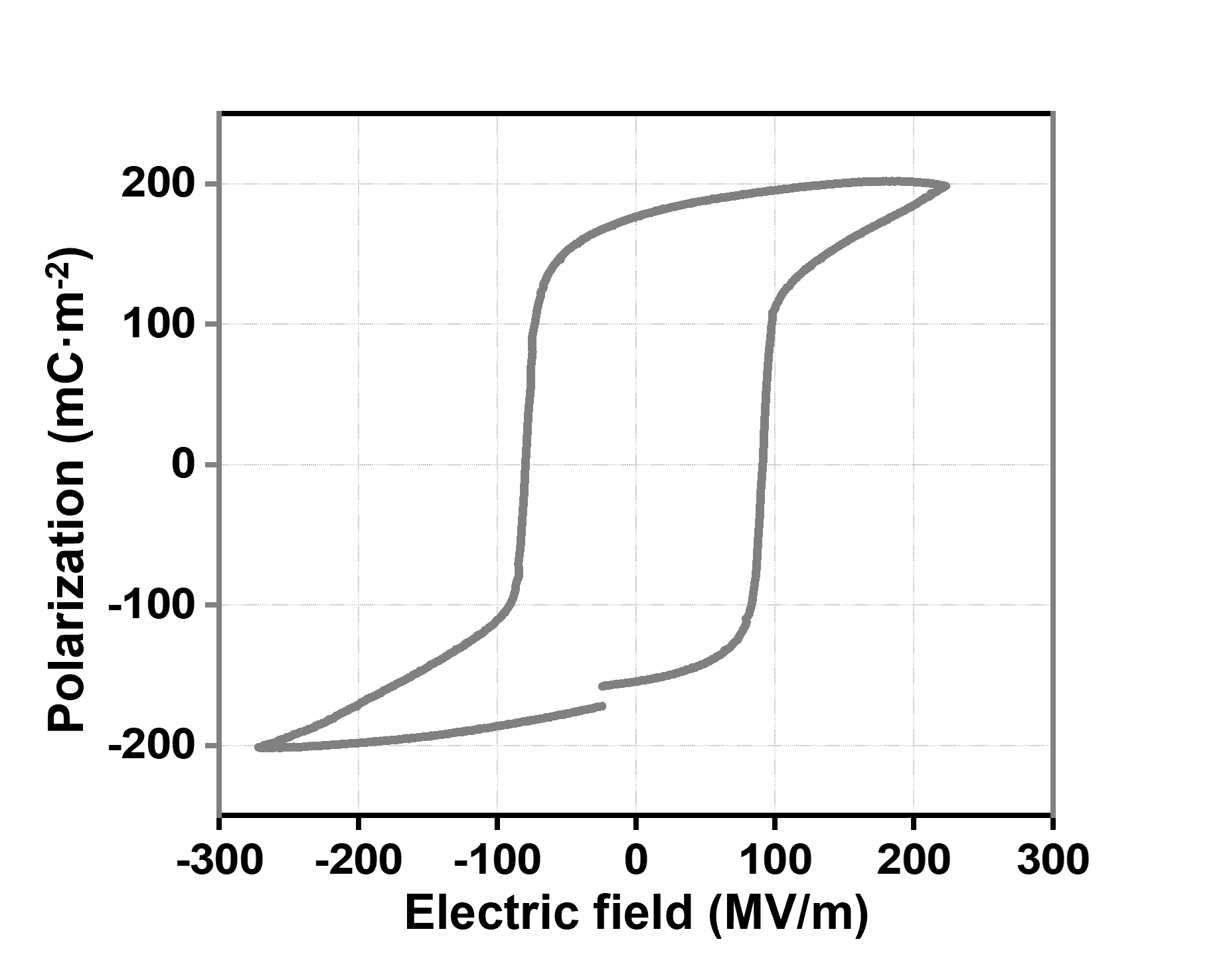


**Figure SI-4│** Polarization-Field (P-E) hysteresis curve for the sample shown in Figure SI-2.


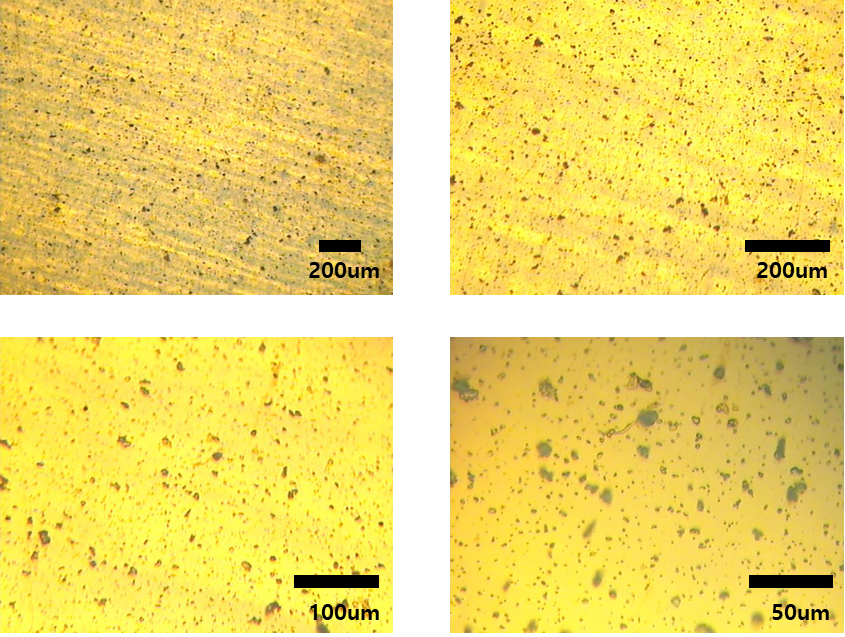


**Figure SI-5│** OM images of the PVDF-coated Al plate after collecting the fine dust. This result shows that PMs of various sizes were adsorbed to the PVDF coated Al plate by electrostatic attraction.


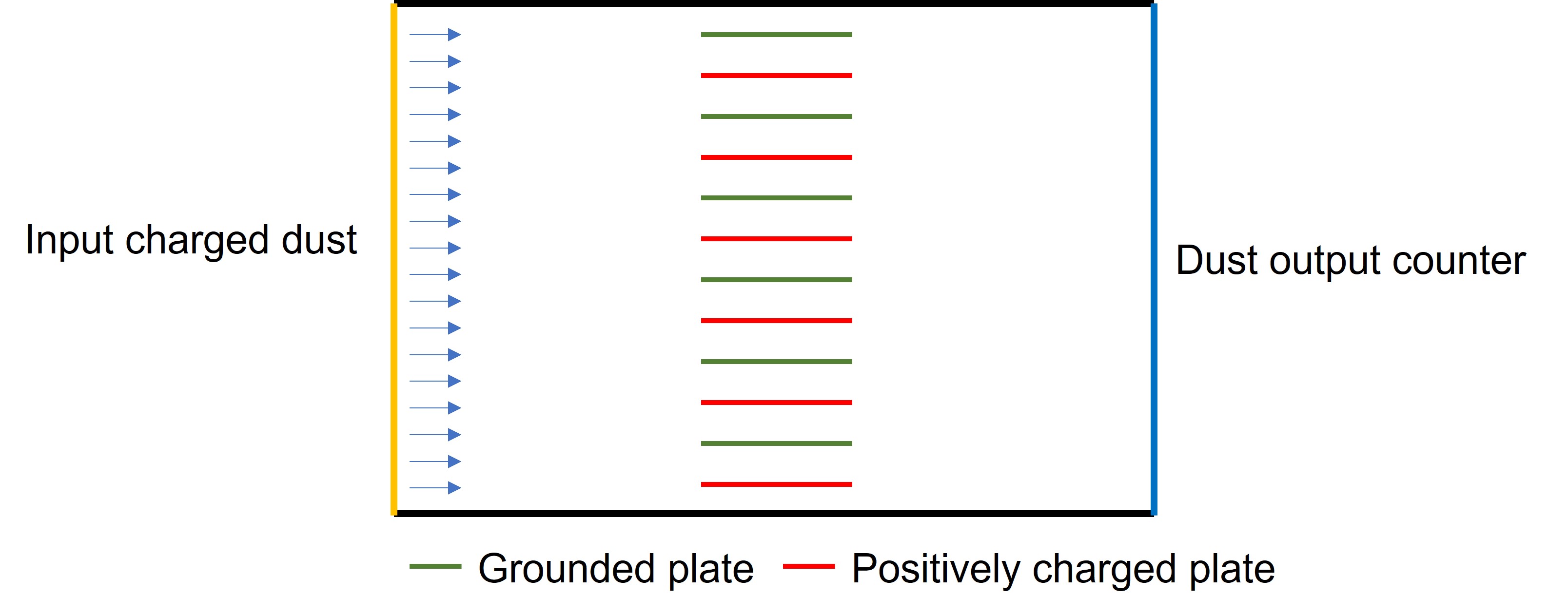


**Figure SI-6│** FEM simulation conditions for collection efficiency of fine dust particles according to surface charge density and flow velocity in the air duct. Bare Al and PVDF-coated Al were placed at intervals of 2 cm in a 20 cm-sized air duct, and bare Al was connected to the ground. 0, 2×10, 4×10, 6×10, and 8×10 C/m^2^ of surface charge density were applied to PVDF-coated Al, and the velocity of fine dust was set to 0, 2, 4, 6, 8, and 10 m/s.


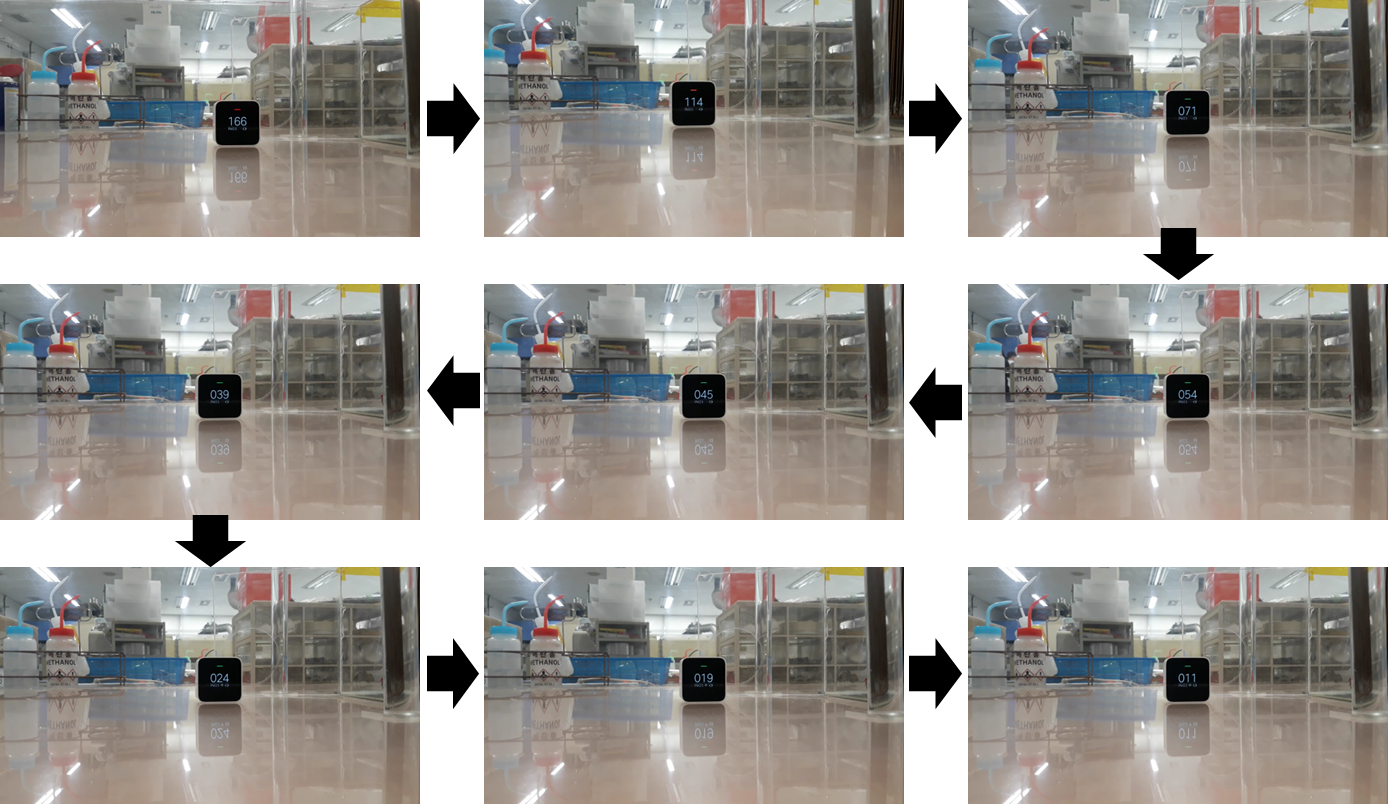


**Figure SI-7│**Experimental data of PM2.5 level that were measured by a dust detector. The PM sensor shows that the PM value decreases significantly from 166 to 11 μg/m^3^ while the TENG drives for 8 min.


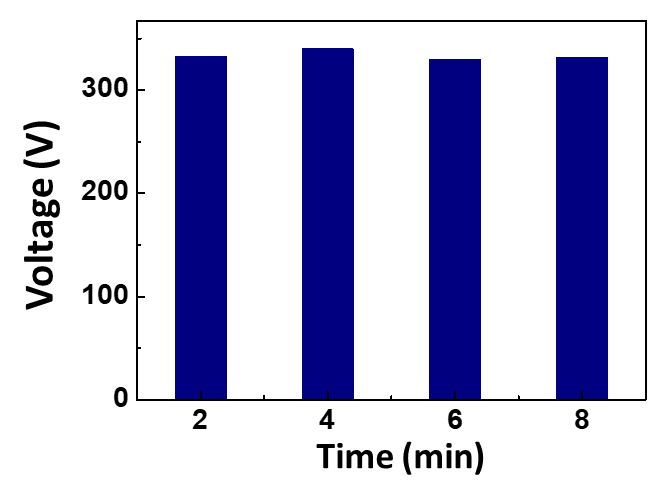


**Figure SI-8 │** Output voltage of TENG for 8 minutes at a load resistance of 40 MΩ.
